# Myeloid cell‐specific mutation of *Spi1* selectively reduces M2‐biased macrophage numbers in skeletal muscle, reduces age‐related muscle fibrosis and prevents sarcopenia

**DOI:** 10.1111/acel.13690

**Published:** 2022-09-13

**Authors:** Ying Wang, Steven S. Welc, Michelle Wehling‐Henricks, Ying Kong, Connor Thomas, Enca Montecino‐Rodriguez, Kenneth Dorshkind, James G. Tidball

**Affiliations:** ^1^ Molecular, Cellular & Integrative Physiology Program University of California Los Angeles California USA; ^2^ CAS Key Laboratory of Quantitative Engineering Biology, Shenzhen Institute of Synthetic Biology, Shenzhen Institute of Advanced Technology Chinese Academy of Sciences Shenzhen China; ^3^ Department of Anatomy, Cell Biology & Physiology Indiana University School of Medicine Indianapolis Indiana USA; ^4^ Indiana Center for Musculoskeletal Health Indiana University School of Medicine Indianapolis Indiana USA; ^5^ Department of Integrative Biology and Physiology University of California Los Angeles California USA; ^6^ Department of Pathology and Laboratory Medicine, David Geffen School of Medicine at UCLA University of California Los Angeles California USA

**Keywords:** aging, macrophage, Sarcopenia, skeletal muscle

## Abstract

Intramuscular macrophages play key regulatory roles in determining the response of skeletal muscle to injury and disease. Recent investigations showed that the numbers and phenotype of intramuscular macrophages change during aging, suggesting that those changes could influence the aging process. We tested that hypothesis by generating a mouse model that harbors a myeloid cell‐specific mutation of *Spi1*, which is a transcription factor that is essential for myeloid cell development. The mutation reduced the numbers of macrophages biased to the CD163+/CD206+ M2 phenotype in muscles of aging mice without affecting the numbers of CD68‐expressing macrophages and reduced the expression of transcripts associated with the M2‐biased phenotype. The mutation did not affect the colony‐forming ability or the frequency of specific subpopulations of bone marrow hematopoietic cells and did not affect myeloid/lymphoid cell ratios in peripheral blood leukocyte populations. Cellularity of most myeloid lineage cells was not influenced by the mutation. The *Spi1* mutation in bone marrow‐derived macrophages in vitro also did not affect expression of transcripts that indicate the M2‐biased phenotype. Thus, myeloid cell‐targeted mutation of *Spi1* influences macrophage phenotype in muscle but did not affect earlier stages of differentiation of cells in the macrophage lineage. The mutation reduced age‐related muscle fibrosis, which is consistent with the reduction of M2‐biased macrophages, and reduced expression of the pro‐fibrotic enzyme arginase. Most importantly, the mutation prevented sarcopenia. Together, our observations indicate that intramuscular, M2‐biased macrophages play significant roles in promoting detrimental, age‐related changes in muscle.

## Graphical abstract

Muscle aging leads to sarcopenia, which is characterized by reductions in muscle mass and muscle fiber size and is also associated with increases in muscle fibrosis. In our investigation, we show that the targeted mutation of the transcription factor Spi1 in myeloid cells, causes a selective reduction in the activation of intramuscular macrophages to an M2‐biased phenotype and that leads to reductions in sarcopenia and fibrosis during muscle aging. These findings show an important role for M2‐biased macrophages in age‐related changes in skeletal muscle.

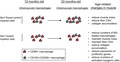

**Abbreviations**
AEC 3: amino‐9‐ethylcarbazole
Arg1: arginase‐1
BMC: bone marrow cell
BMDM: bone marrow‐derived macrophage
BMT: bone marrow transplantation
CD: cluster of differentiation
CMP: common myeloid progenitor
Col1α1: collagen 1, alpha‐1 chain
Col3α1: collagen3, alpha‐1 chain
CSA: cross‐sectional area
Ct: cycle threshold
DMEM: Dulbecco’s modified Eagle medium
DPBS: Dulbecco’s phosphate buffered saline
FACS: fluorescence‐activated cell sorting
FBS: fetal bovine serum
FITC: fluorescein isothiocyanate
Fizz‐1: found in inflammatory zone one
floxed: flanked loxP
GM‐CSF: granulocyte‐macrophage colony stimulating factor
GMP: granulocyte‐macrophage progenitor cell
HSC: hematopoietic stem cell
Ig: immunoglobin
IGF‐1: insulin‐like growth factor‐1
IL‐10: interleukin‐10
IL‐3: interleukin‐3
IL‐4: interleukin‐4
loxP: locus of x‐over P1
Ly6C: lymphocyte antigen 6 complex, locus C1
LysM: lysine motif
lyz2: lysozyme 2
M‐CSF: macrophage colony‐stimulating factor
MEM: minimal essential medium
MEP: megakaryocyte‐erythroid progenitor cell
min: minute
Mrc1: mannose receptor C‐type 1
My‐HSC: myeloid‐biased hematopoietic stem cell
MyoD: myoblast determination protein 1
NK: natural killer
Nos1: nitric oxide synthase‐1
Pax7: paired box 7
PBS: phosphate‐buffered saline
PE: phycoerythrin
PerCp: peridinin chlorophyll protein‐cyanine
PPARδ: peroxisome proliferation activator receptor delta
PPIA: peptidylprolyl isomerase A
QPCR: quantitative polymerase chain reaction
quad: quadriceps
Retnla: resistin‐like alpha
RPMI: Roswell Park Memorial Institute
Sca: stem cell antigen
SEM: standard error of the mean
Spi1: spleen focus forming virus (SFFV) proviral integration oncogene
TA: tibialis anterior
TCR: T‐cell receptor
TGFβ: transforming growth factor beta
TPT1: translationally controlled tumor protein 1

## 1 INTRODUCTION

Aging of skeletal muscle causes an inevitable and relentless loss of muscle mass and an increase in muscle fibrosis that reduce function and quality of life. Many of the age‐related changes are attributable to senescence of the muscle fibers themselves. For example, old muscle fibers show reductions in protein synthesis and increases in proteolysis that contribute to loss of muscle mass and function during aging (Combaret et al., 2009; Pluskal et al., 1984). In addition, age‐related increases in muscle fibrosis are affected by changes in muscle stem cells, called satellite cells, as their myogenic capacity declines and their fibrogenic capacity increases (Brack et al., 2007). However, these age‐related changes in muscle cells are also influenced by aging of non‐muscle cells that play key regulatory roles in maintaining normal muscle homeostasis.

Because the immune system influences the function of every tissue, aging of the immune system can affect cell functions throughout the body. A fundamental change in the immune system of aging animals occurs at the earliest stages of hematopoietic development. Differentiating hematopoietic stems cells (HSCs), which give rise to almost all blood cells, experience reduced lymphoid potential and a skewing towards the myeloid lineage (Dorshkind et al., 2020). This age‐related, myeloid bias is reflected in the periphery where the numbers of mature myeloid cells increase in the circulation (Della Bella et al., 2007). The elevation in their number coincides with an increase in frailty in the elderly, which may have important influences on muscle health because myeloid cells, especially macrophages, play powerful regulatory roles in muscle growth (Tidball, 2017).

The regulatory influence of macrophages varies according to their activation. Although macrophages exist on a continuous spectrum of functional states (Murray et al., 2014), one end of the spectrum has been designated as the M1 phenotype, which is activated by proinflammatory molecules; at the other end of the spectrum, M2 macrophages are activated by anti‐inflammatory cytokines (Mills et al., 2000). Both phenotypes play beneficial roles in muscle repair and regeneration. M1‐biased macrophages can increase proliferation of satellite cells, which contributes to muscle regeneration (Bencze et al., 2012). Macrophages biased toward the M2 phenotype are also pro‐regenerative through their release of factors that increase muscle growth (Tonkin et al., 2015; Wehling‐Henricks et al., 2018) and the production of factors that can increase connective tissue production, which provides a framework for tissue repair (Mills, 2001).

Our previous investigations indicate that some age‐related changes of muscle are attributable to changes in intramuscular macrophages. For example, expression of an *Nos1* transgene in murine muscle reduced the age‐related accumulation of M2‐biased pro‐fibrotic macrophages and prevented the age‐related accumulation of intramuscular collagens (Wang et al., 2015). Similarly, aging human muscle experiences an accumulation in M2‐biased macrophages and increased fibrosis (Csapo et al., 2014; Cui et al., 2019), although other investigators report no increase in intramuscular collagen in aging humans (Haus et al., 2007). In addition, transplantation of young bone marrow cells into adult mice reduced sarcopenia and muscle fibrosis in the recipients as they aged (Wang et al., 2019), implicating aging of the immune system with muscle loss and fibrosis.

Those observations suggest a link between M2‐biased macrophages and fibrosis in aging muscle. However, the experimental interventions that were used to affect the numbers, phenotype or age of myeloid cells in aging muscle would have also influenced other cell types in that tissue. For example, immune cells that include CD8+ cytotoxic T‐cells (Zhang et al., 2014) and regulatory T‐cells (Burzyn et al., 2013; Wang et al., 2015) are also present in skeletal muscle where they can influence myogenesis; the functions of those cells could also be affected in mice receiving heterochronic bone marrow transplantation (BMT).

In the present investigation, we generated a mouse line in which the *Spi1* gene is mutated in myeloid lineage cells to determine the regulatory role of *Spi1* in myeloid cells that are present in aging muscle. *Spi1* encodes the transcription factor PU.1, which is essential in determining the differentiation fate of hematopoietic cells (DeKoter et al., 2007). High levels of expression of *Spi1* are required for differentiation of common myeloid progenitors (CMPs) into mature monocytes/macrophages (Lichanska et al., 1999). Although germ line deletion of *Spi1* results in almost complete hematopoietic failure and death of mice in utero or within days after birth (McKercher et al., 1996; Scott et al., 1994), we reasoned that macrophage‐deficient mice could be generated by crossing *Spi1* ^*floxed*^ mice with L*ysM* ^*Cre*^ mice, in which Cre recombinase is driven by the promoter of the *lysozyme2* gene (*lyz2*) that is expressed exclusively in myeloid cells. A previous study showed that crossing *LysM* ^*Cre*^ mice with mice with loxP‐flanked target genes results in high deletion efficiency of target genes in mature macrophages (Clausen et al., 1999).

Our findings reveal unexpected effects of *lyz2*‐driven deletion of *Spi1*. We found that the mutation did not reduce the numbers of differentiated, CD68+ macrophages in aging muscle; instead the mutation reduced the numbers of intramuscular macrophages that were activated to the CD163+/CD206+, M2‐biased phenotype. This outcome provided us with a tool for assessing the role of M2‐biased macrophages in muscle aging and allowed us to validate the important role of that macrophage phenotype in sarcopenia and fibrosis of aging muscle.

## 2 RESULTS

### 2.1 *Spi1* mutation driven by the *lyz2* promoter reduces the number of M2‐biased macrophages in muscle

**FIGURE 1:**
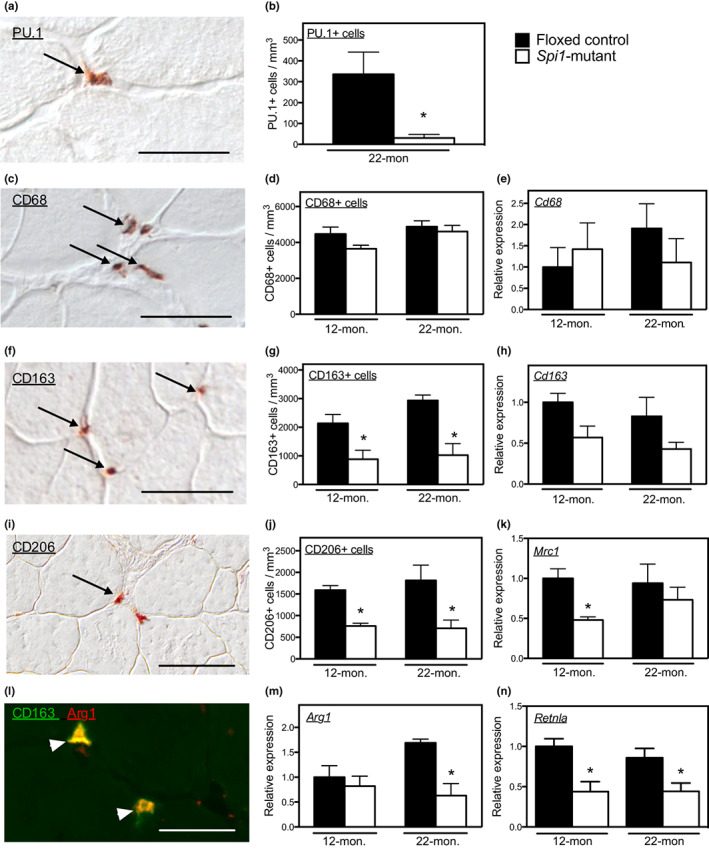
Myeloid cell‐specific mutation of *Spi1* reduced M2‐biased macrophage numbers in muscle. (a) PU.1+ cells (arrow) in 22‐month‐old floxed‐control quadriceps. Scale bar = 30 μm. (b) Number of PU.1+ cells per unit muscle volume in quadriceps muscle cross‐sections from 22‐month‐old mice. (c) CD68+ cells (arrows) in 22‐month‐old floxed‐control quadriceps. Scale bar = 50 μm. (d) Number of CD68+ macrophages in *Spi1*‐mutant and floxed‐control mice at 12 and 22 months. (e) Expression levels of *Cd68* in *Spi1*‐mutant and floxed‐control mice at 12 and 22 months. (f) Quadriceps muscle cross‐section from a 22‐month‐old *Spi1*‐mutant mouse stained for CD163. Bars = 50 μm. (g) Numbers of CD163+ macrophages in 12‐ and 22‐month‐old *Spi1*‐mutant mice and floxed‐control mice. (h) *Cd163* expression showed an insignificant trend of decrease in *Spi1*‐mutant mice compared to age‐matched floxed‐control mice at 12 and 22 months old. (i) Quadriceps muscle cross‐section from a 22‐month‐old *Spi1*‐mutant mouse stained for CD206. Bars = 50 μm. (j) Numbers of CD206+ macrophages in 12‐ and 22‐month‐old *Spi1*‐mutant mice compared to age‐matched floxed‐control mice. (k) *Mrc1* expression in 12‐ and 22‐month‐old *Spi1*‐mutant mice compared to age‐matched floxed‐control mice. (l) Quadriceps muscle cross‐section from a 22‐month‐old floxed‐control mouse stained for CD163 (488 nm; green) and Arg1 (594 nm; red). Arrowheads show double‐labeled cells (yellow). Bars = 50 μm. (m) *Arg1* expression in 12‐ and 22‐month‐old *Spi1*‐mutant mice compared to age‐matched floxed‐control mice. (n) *Retnla* expression in 12‐ and 22‐month‐old *Spi1*‐mutant mice compared to age‐matched floxed‐control mice. For all panels, * indicates significant difference from age‐matched, floxed‐control group assayed by two‐tailed *t*‐test at *p* < 0.05. *N* = 4–5 per data set.

*LysM* ^Cre^/*Sfpi1* ^Lox^ mice (referred to as *Spi1*‐mutants hereafter) were analyzed up to 22 months of age, which revealed that they exhibited normal survival and no obvious morphological or behavioral differences compared to their *LysM* ^wildtype^/*Sfpi1* ^Lox^ littermates (referred to as floxed‐controls hereafter). We assayed for *Spi1*‐expressing cells in quadriceps muscles of 22‐months‐old mice by immunohistochemistry and found that *Spi1*‐mutant mice had more than a 90% reduction in PU.1+ cells compared to age‐matched floxed‐control mice (Figure 1a,b).

Because intramuscular macrophages consist of heterogeneous subpopulations (Tidball, 2017), we examined the effect of *Spi1* mutation on subpopulations of intramuscular macrophages. Surprisingly, we found that the number of CD68+ macrophages did not differ in quadriceps of *Spi1*‐mutant mice compared to floxed‐control mice at 12‐ or 22‐months of age (Figure 1c,d) and *Cd68* mRNA expression did not differ between floxed‐control or *Spi1*‐mutant mice at 12 and 22 months of age (Figure 1e). Those data show that myeloid‐specific mutation of *Spi1* did not prevent the differentiation of monocytes/macrophages in muscle. However, the numbers of CD163+ M2‐biased macrophages were significantly lower in 12‐ and 22‐month‐old *Spi*‐mutant mice compared to age‐matched floxed‐control mice (Figure 1f,g). Our QPCR results also showed a trend for reduced expression of *Cd163* in quadriceps of *Spi1*‐mutant mice compared to floxed‐control mice at both 12 and 22 months (Figure 1h). Because we found that ~73% of CD68+ macrophages in muscles of floxed‐control mice expressed CD163 but only ~27% of CD68+ macrophages in *Spi1*‐mutant mice expressed CD163 (Figure S1a,b) while *Spi1* mutation did not affect total number of CD68+ macrophages (Figure 1d), the findings indicate that the mutation reduced the activation of macrophages to a CD163+, M2‐biased phenotype without affecting total macrophage numbers in aging muscle.

We also assayed the expression of CD206, another marker of M2‐biased macrophages in muscle (Vidal et al., 2008; Villalta et al., 2011; Wang et al., 2014) and found that CD206+ macrophage numbers were reduced by *Spi1* mutation in both 12‐ and 22‐month‐old muscles (Figure 1i,j). *Mrc1* (which encodes CD206), also showed significantly lower expression in *Spi1*‐mutant mice compared to floxed‐controls at 12‐months of age and a trend for lower expression in mutant mice at 22‐months of age (Figure 1k). Notably, some CD163+ intramuscular macrophages did not express detectible levels of CD206 (Figure S1c), showing that expression of CD163 and CD206 do not indicate an identical population of M2‐biased macrophages in muscle.

Although the decreases in CD163+ and CD206+ cell numbers could reflect impaired macrophage differentiation into the M2 phenotype, they may also reflect the downregulation of CD163 and CD206 expression in M2‐biased macrophages because the genes encoding CD163 and CD206 have PU.1 binding sites in their promoter regions and their promoter activity is directly regulated by PU.1 (Eichbaum et al., 1997; Ritter et al., 1999). We tested whether other transcripts that reflect macrophage activation to an M2‐biased phenotype that were not direct targets of PU.1 were also affected by the mutation. We first confirmed that CD163+, intramuscular macrophages expressed arginase‐1 (Figure 1l) and then found through QPCR analysis that expression of *Arg1* (which encodes arginase‐1) was significantly reduced by myeloid‐specific mutation of *Spi1* in 22‐month‐old muscles (Figure 1m). We also observed that expression of *Retnla* (which encodes Fizz‐1, another M2 phenotypic marker) was decreased in 12‐ and 22‐month‐old *Spi1*‐mutant mice compared to age‐matched floxed‐control (Figure 1n). These data indicate that myeloid cell‐specific mutation of *Spi1* selectively reduced M2‐biased macrophage numbers in muscles at both 12 and 22 months of age.

### 2.2 Myelopoiesis is intact in *Spi1*‐mutant mice

**FIGURE 2:**
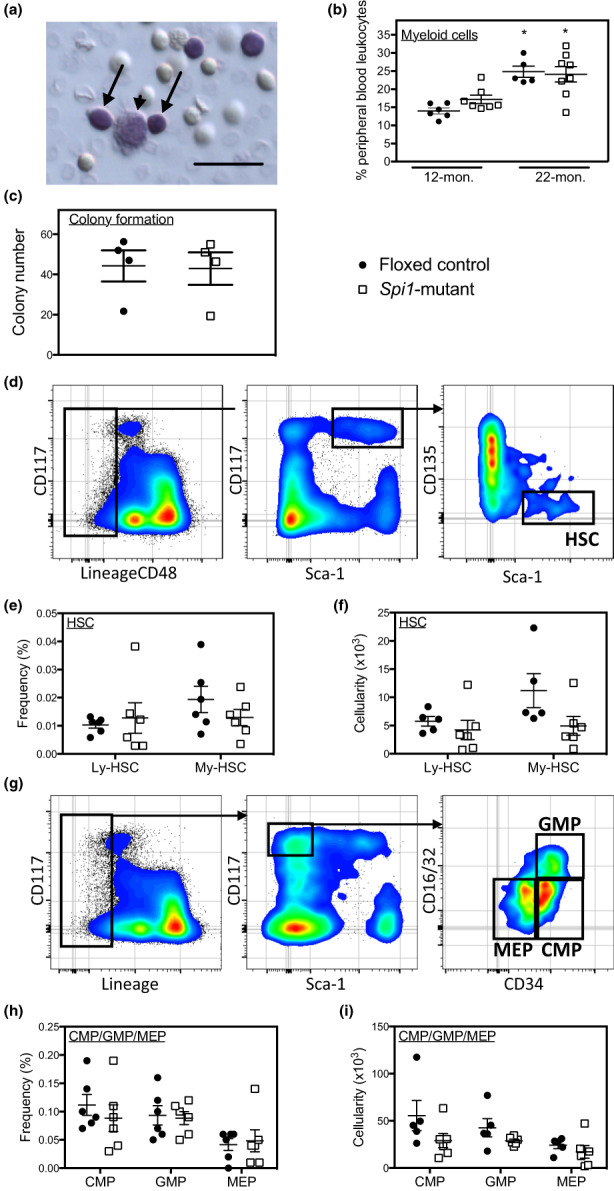
Hematopoietic function and the frequencies and cellularities of subpopulations of hematopoietic and immune cells in floxed‐control and *Spi1*‐mutant mice. (a) Cytospin preparation of whole blood collected from 22‐month *Spi1*‐mutant mouse showing representative lymphoid (arrows) and myeloid (arrowhead) cells. Scale bar = 30 μm. (b) the proportion of myeloid cells in peripheral blood leukocytes in floxed‐control and *Spi1*‐mutant mice at 12 and 22 months old. * indicates significant difference from 12‐month‐old mice of the same genotype. (c) the colony‐forming ability of BMCs in 22‐month‐old floxed‐control and *Spi1*‐mutant mice. (d) Strategy used for resolution of Ly‐HSCs and my‐HSCs from BMCs of 22‐month‐old floxed‐control and *Spi1*‐mutant mice. (e) the frequencies of Ly‐HSCs and my‐HSCs in 22‐month‐old floxed‐control and *Spi1*‐mutant mice. (f) the cellularities of Ly‐HSC and my‐HSC in 22‐month‐old floxed‐control and *Spi1*‐mutant mice. (g) Strategy used for resolution of CMPs, GMPs and MEPs from BMCs of 22‐month‐old floxed‐control and *Spi1*‐mutant mice. (h) the frequencies of CMPs, GMPs and MEPs in 22‐month‐old floxed‐control and *Spi1*‐mutant mice. (i) the cellularities of CMPs, GMPs and MEPs in 22‐month‐old floxed‐control and *Spi1*‐mutant mice.

We assayed whether the *Spi1*‐mutation would disrupt myelopoiesis by testing for effects of the mutation on the proportion of peripheral blood leukocytes that exhibited myeloid cell morphology but found no differences in the proportion of circulating leukocytes that was comprised of myeloid cells in *Spi1*‐mutants and floxed‐controls at either 12‐ or 22‐months of age (Figure 2a,b). However, we did observe that aging similarly increased the proportion of peripheral blood leukocytes that were comprised of myeloid cells in both *Spi1*‐mutant and floxed‐control mice (Figure 2b).

Because *lyz2* is expressed in relatively mature myeloid cells and not progenitors, we expected that differences in primary myelopoiesis would not explain the reduced number of M2‐biased macrophages in *Spi1* mutants compared to floxed‐control mice. We first quantified the number of myeloid colonies generated in semisolid medium by culturing BMCs with a cocktail of myelopoietic cytokines and found no differences in the colony‐forming ability of BMCs isolated from *Spi1*‐mutant and floxed‐control mice (Figure 2c). Those findings were then confirmed by more detailed FACS analyses, which showed there were no significant differences in the frequency and number of myeloid‐biased HSCs (My‐HSCs; Figure 2d–f), CMPs, granulocyte‐macrophage progenitors (GMPs) or megakaryocyte‐erythroid progenitors (MEPs) in *Spi1*‐mutant and floxed‐control mice (Figure 2g–i). Taken together, these results indicate that *LysM* ^Cre^ driven deletion of *Spi1* has minimal effects on primary myelopoiesis.

### 2.3 Myeloid cell‐specific *Spi1* mutation does not obviate induction of the M2 phenotype in bone marrow‐derived macrophages in vitro

**FIGURE 3:**
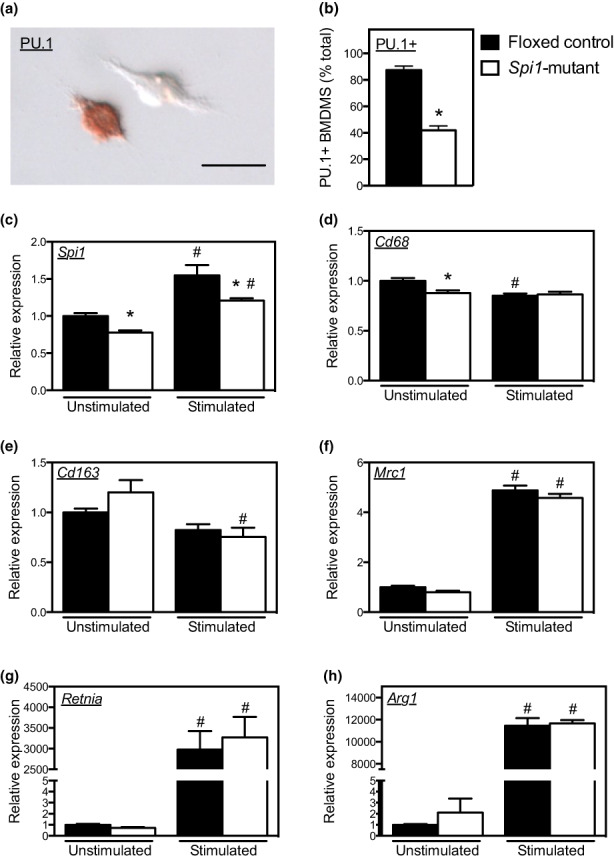
Expression levels of *Spi1* and macrophage phenotypic markers in BMDMs of 12‐month‐old floxed‐control and *Spi1*‐mutant mice without stimulation and with M2 polarization (stimulated). (a) *Spi1‐*mutant BMDMs cultured on coverslips and labeled with anti‐PU.1, showing one BMDM expressing high levels of nuclear PU.1 (red) and one BMDM with undetectable nuclear PU.1. Bar = 10 μm. (b)*Spi1‐*mutants show a smaller proportion of BMDMs that express detectible, nuclear PU.1, assayed by immunohistochemistry. (c) *Spi1* expression in floxed‐control and *Spi1*‐mutant BMDMs without or with M2 polarization. (d) *Cd68* expression in floxed‐control and *Spi1*‐mutant BMDMs without or with M2 polarization. (e) *Cd163* expression in floxed‐control and *Spi1*‐mutant BMDMs without or with M2 polarization. (f) *Mrc1* expression in floxed‐control and *Spi1*‐mutant BMDMs without or with M2 polarization. (g) *Retnla* expression in floxed‐control and *Spi1*‐mutant BMDMs without or with M2 polarization. (h) *Arg1* expression in floxed‐control and *Spi1*‐mutant BMDMs without or with M2 polarization. For all panels, * indicates significant difference between genotypes with the same treatment. # indicates significant differences between treatments with the same genotype. *N* = 4–5 per data set.

Because muscles of *Spi1*‐mutant mice showed reduced numbers of CD163+ and CD206+ macrophages and reduced levels of expression of transcripts associated with the M2‐biased macrophage phenotype in vivo, we assayed whether myeloid cells derived from the bone marrow of *Spi1*‐mutant mice showed an intrinsic defect in responding to growth factors and cytokines that promote the M2‐biased macrophage phenotype. First, we confirmed that the *Spi1* mutation reduced the number of bone marrow‐derived macrophages (BMDMs) that expressed detectible levels of PU.1 (Figure 3a,b). Our QPCR analysis showed that the expression of markers of macrophage differentiation and activation (*Cd68*, *Cd163*, *Mrc1*, *Retnla*, *Arg1*) in *Spi1*‐mutant BMDMs differed little from levels of expression of those markers in floxed‐control BMDMs (“unstimulated” conditions in Figure 3c–h). These results indicate that reduced expression of *Spi1* caused by a lysozyme 2 driven deletion of *Spi1* did not impair macrophage differentiation into an M2 phenotype under in vitro stimulation conditions.We then assayed whether the Th2 cytokines IL‐4 and IL‐10 affected expression of *Spi1* or M2‐related transcripts in *Spi1*‐mutant and floxed‐control BMDMs. IL‐4 and IL‐10 can each promote the M2‐biased phenotype in intramuscular macrophages and can increase the expression of *Cd163* and *Cd206* (Buechler et al., 2000; Sulahian et al., 2000; Villalta et al., 2011). IL‐4/IL‐10 stimulation of BMDMs caused elevated *Spi1* expression in both *Spi1*‐mutants and floxed‐controls, although the level of expression in stimulated, mutant BMDMs was less than controls (Figure 3c). IL‐4/IL‐10 stimulation slightly decreased *Cd68* expression in floxed‐control BMDMs but the expression of *Cd68* in *Spi1*‐mutant BMDMs was not significantly affected by the cytokines (Figure 3d). We also observed that IL‐4/IL‐10 stimulation did not elevate expression of *Cd163* in either mutant or control BMDMs and that the mutation did not affect the level of *Cd163* expression in either the unstimulated or the IL‐4/IL‐10 stimulated groups (Figure 3e). In contrast to the lack of induction of *Cd163* by IL‐4/IL‐10 in BMDMs, we observed a large induction of expression of *Mrc1*, *Retnla* and *Arg1* (Figures 3f–h). However, their expression levels were not different in *Spi1*‐mutant and floxed‐control BMDMs within the same treatment conditions (Figure 3f–h).

Together, these data demonstrate that hematopoietic cells from *Spi1* mutant mice differentiate normally to M2‐biased macrophages in vitro but show a reduction of CD163+ and CD206+ macrophages and reduced expression of M2 phenotypic markers in aging muscle of *Spi1*‐mutant mice (Figure 1f–n). This indicates that the reduction in M2‐biased macrophage numbers in *Spi1*‐mutant muscles is not intrinsic to myeloid lineage cells, but instead may be influenced by the environment in which differentiation and activation occur.

### 2.4 Myeloid cell‐specific mutation of *Spi1* reduced connective tissue accumulation in old muscle

**FIGURE 4:**
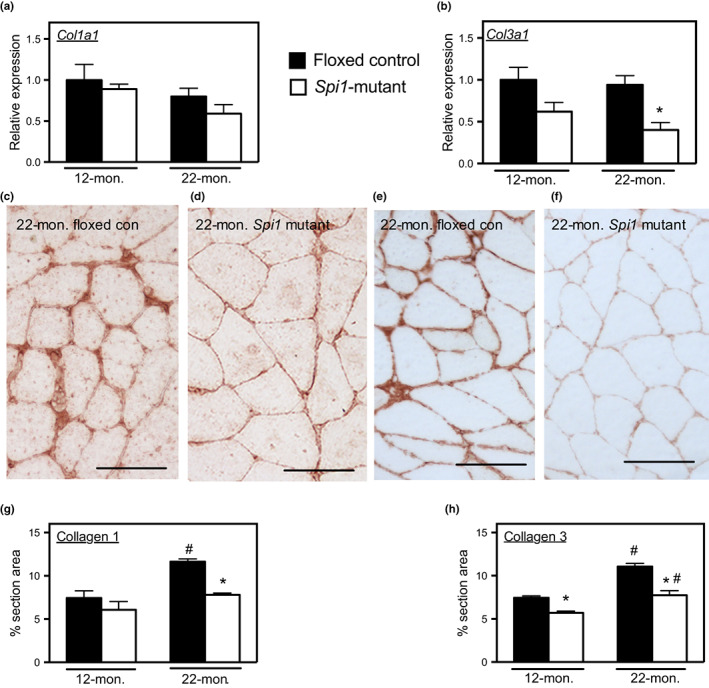
Myeloid cell specific mutation of *Spi1* reduced the accumulation of connective tissue in aging muscles. (a) *Col1a1* expression in 12‐ and 22‐month‐old floxed‐control and *Spi1*‐mutant mice. (b) *Col3a1* expression in 12‐ and 22‐month‐old floxed‐control and *Spi1*‐mutant mice. (c and d) immunohistochemistry of collagen type I distribution (reddish‐brown) in sections of quadriceps muscle from 22‐month‐old floxed‐control (c) *or Spi1‐*mutant (d). Scale bars = 50 μm. (e and f) immunohistochemistry of collagen type III distribution (reddish‐brown) in sections of quadriceps muscle from 22‐month‐old floxed control (e) *or* Spi1 mutant (f). Scale bars = 50 μm. (g) the proportion of section area consisting of collagen type I in floxed‐control and *Spi1*‐mutant mice at 12 and 22 months. (h) the proportion of section area consisting of collagen type III in floxed‐control and *Spi1*‐mutant mice at 12 and 22 months. For all panels, * indicates significant difference in age‐matched groups between genotypes. # indicates significant differences between ages of same genotype. *N* = 5 per data set.

We showed in a previous investigation that the age‐related shift toward greater numbers of CD163+ M2‐biased macrophages in muscle is associated with increased muscle fibrosis (Wang et al., 2015). Because *Spi1*‐mutant mice showed reduced numbers of M2‐biased macrophages in both adult and old muscles compared to age‐matched floxed‐control mice, we tested whether this reduction in CD163+ and CD206+ macrophages affected muscle fibrosis during aging. Expression of *Col1a1* (which encodes the alpha 1 chain of collagen type I) and *Col3a1* (which encodes the alpha 1 chain of collagen type III) did not change significantly between 12 and 22 months in floxed‐control or *Spi1*‐mutant mice (Figure 4a,b). However, the expression of *Col3a1* was significantly lower in 22‐month‐old *Spi1*‐mutant mice compared to age‐matched floxed‐control mice (Figure 4b). The volume fraction of muscle occupied by collagen type I and type III increased between 12 and 22 months of age (Figures 4c–h). Furthermore, we observed that myeloid‐specific mutation of *Spi1* prevented the age‐related accumulation of collagen type I and significantly reduced the accumulation of collagen type III during aging (Figure 4g,h). These findings indicate that the increase of M2‐biased macrophages during aging contributes to age‐related muscle fibrosis and that the myeloid cell‐specific *Spi1* mutation can reduce the accumulation of connective tissue in aging muscle by decreasing macrophages that are biased toward the M2 phenotype.

### 2.5 Myeloid cell‐specific mutation of *Spi1* prevented sarcopenia

**FIGURE 5:**
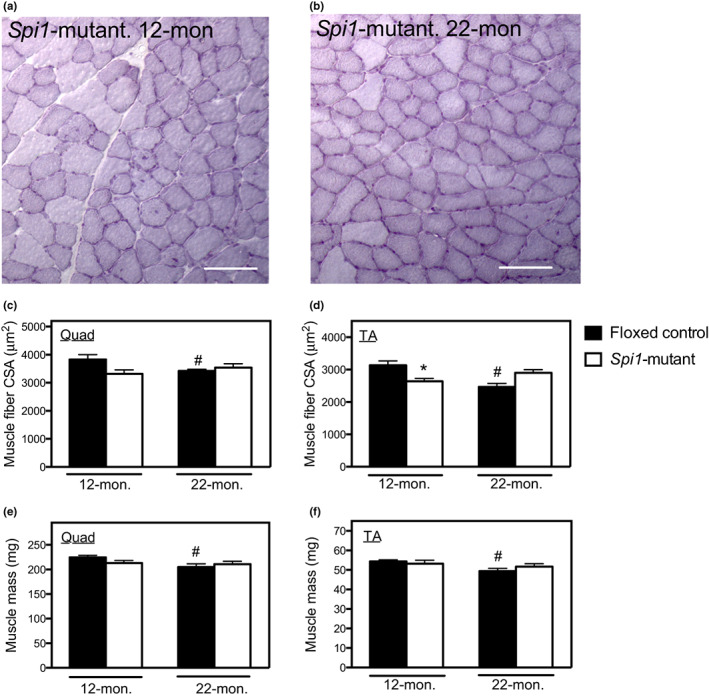
Myeloid cell‐specific mutation of *Spi1* prevents sarcopenia. (a and b) images of hematoxylin‐stained cross‐sections of a quadriceps muscle from a 12‐month‐old *Spi1*‐mutant mouse (a) or from a 22‐month‐old *Spi1*‐mutant mouse (b). Bars = 60 μm. (c and d) muscle fiber cross‐sectional area (CSA) of quadriceps (quad; panel c) and TA (panel d) in floxed‐control mice and *Spi1*‐mutant mice at 12 and 22 months of age. (e and f) masses of quad (e) and TA (f) muscles in floxed‐control mice and *Spi1*‐mutant mice at 12 and 22 months of age. For panels c–f, * indicates significant difference in age‐matched groups between genotypes. # indicates significant differences between ages of same genotype.

We then tested whether the myeloid cell‐specific mutation affected sarcopenia by quantifying the cross‐sectional area (CSA) of muscle fibers in 12‐ and 22‐month‐old muscles. CSA of quadriceps decreased significantly between 12 and 22 months in floxed‐control mice, but not in *Spi1*‐mutant mice (Figure 5a–c). Similarly, CSA of TAs decreased significantly between 12 and 22 months in floxed‐control mice but did not decrease during aging in *Spi1*‐mutant mice (Figure 5d).

Notably, muscle fiber CSAs in 12‐months‐old TAs were smaller in *Spi1* mutants than in floxed‐control mice (Figure 5d). However, because mice would not be sarcopenic at 12 months old, the smaller fiber size in *Spi1*‐mutant mice at this age would be attributable to reduced fiber growth in younger mice; M2‐biased macrophages are sources of growth factors including IGF‐1 and Klotho that increase muscle fiber growth (Dumont & Frenette, 2010; Tonkin et al., 2015; Wehling‐Henricks et al., 2018).

Similar to the effects of *Spi1* mutation on reductions in fiber CSA during aging, *Spi1* mutants experienced no loss of muscle mass in quadriceps or TAs between 12 and 22 months old, in contrast to floxed‐control mice in which both muscles showed significant, age‐related mass loss (Figure 5e,f). Together, these data show that myeloid cell‐specific mutation of *Spi1* prevented sarcopenia in mice between 12 and 22 months old.

### 2.6 Myeloid cell‐specific mutation of *Spi1* reduced satellite cell activation in adult and aging muscles

**FIGURE 6:**
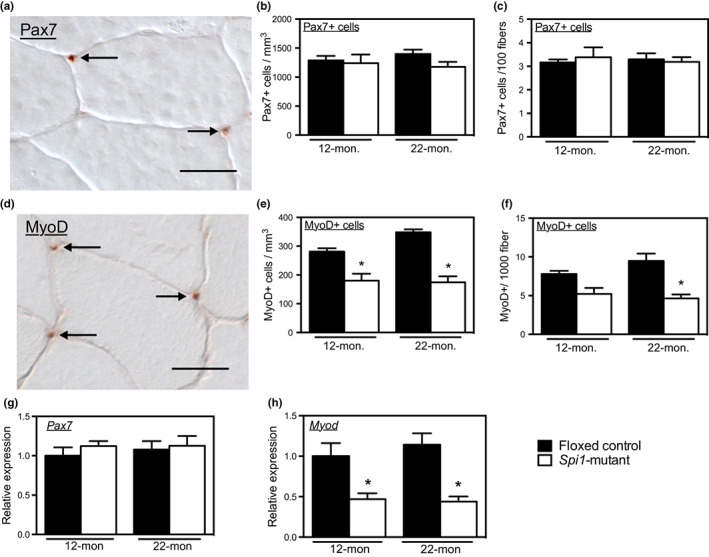
Myeloid cell‐specific mutation of *Spi1* reduced *Myod* expression and reduced MyoD+ cell numbers in 12‐ and 22‐month‐old muscles. (a) Pax7+ cells (arrows) in 22‐month‐old floxed‐control quadriceps. Scale bar = 30 μm. (b) the numbers of Pax7+ cells per unit muscle volume in floxed‐control and *Spi1*‐mutant muscles at 12 and 22 months of age. (c) the numbers of Pax7+ cells per 100 muscle fibers in floxed‐control and *Spi1*‐mutant muscles at 12 and 22 months of age. (d) MyoD+ cells (arrows) in 22‐month‐old floxed‐control quadriceps. Scale bar = 30 μm. (e) the numbers of MyoD+ cells per unit muscle volume in floxed‐control and *Spi1*‐mutant muscles at 12 and 22 months of age. (f) the numbers of MyoD+ cells per 1000 muscle fibers in floxed‐control and *Spi1*‐mutant muscles at 12 and 22 months of age. (g) *Pax7* expression in floxed‐control and *Spi1*‐mutant muscles at 12 and 22 months of age. (h) *Myod* expression in floxed‐control and *Spi1*‐mutant muscles at 12 and 22 months of age. For all panels, * indicates significant difference in age‐matched groups between genotypes. *N* = 5 per data set.

We tested whether reducing M2‐biased macrophage numbers or activation by myeloid‐cell‐specific mutation of *Spi1* affected satellite cell numbers in aging muscle by assaying for the numbers of muscle cells that expressed the myogenic transcription factors Pax7 and MyoD. Pax7 is expressed by quiescent satellite cells or by recently activated satellite cells that have the potential to return to the reserve population of quiescent satellite cells (Zammit et al., 2006). MyoD is expressed by recently activated satellite cells that have the potential to withdraw from the cell cycle and proceed through terminal differentiation (Smith et al., 1994). Although the *Spi1* mutation did not affect the number of Pax7+ satellite cells per unit muscle volume or per 100 muscle fibers in 12‐ and 22‐month‐old mice (Figure 6a–c), the number of activated satellite cells that expressed MyoD was significantly less in *Spi1*‐mutant mice compared to floxed‐control mice at both 12‐ and 22‐ months old (Figure 6d–f). Furthermore, expression of *Pax7* was not affected by the *Spi1* mutation in 12‐ and 22‐month‐old muscles (Figure 6g), although *Myo*d expression was significantly reduced in 12‐ and 22‐month‐old muscles of *Spi1*‐mutant mice compared to age‐matched floxed‐control mice (Figure 6h). Together, these data show that the *Spi1* mutation in myeloid cells influenced the activation of myogenic cells, but not their number, at least at the ages examined in the present investigation. Thus, M2‐biased macrophages may contribute to sarcopenia by affecting satellite cell activation.

## 3 DISCUSSION

In the present study, we demonstrate that the number of macrophages in aging muscle that are biased to the CD163+/CD206+ M2 phenotype is selectively and significantly reduced by a myeloid cell‐specific deletion of *Spi1* without affecting total numbers of CD68+ macrophages. Thus, diminished expression of *Spi1* in myeloid cells in vivo affects macrophage phenotype in muscle, but does not prevent the differentiation of myeloid lineage cells into macrophages. Importantly, this specific perturbation of M2‐biased macrophage numbers was sufficient to reduce sarcopenia and fibrosis of aging muscle. Although previous studies showed that interventions that reduced the numbers of macrophages in aging muscle also produced reductions in connective tissue accumulation (Wang et al., 2015), the experimental approaches used in those studies could have affected non‐myeloid cell populations with unknown roles in muscle aging. Our previous findings also showed that the severity of sarcopenia is affected by aging of the immune system (Wang et al., 2019). However, that investigation did not identify which specific, aging immune cells were responsible for increasing sarcopenia. We now show that reducing numbers of intramuscular, M2‐biased macrophages significantly reduces degenerative changes that occur in aging muscle.

The results of our investigation include several unexpected outcomes. First, the data demonstrate that M2‐biased macrophages can exert detrimental effects on homeostasis of non‐injured muscle. This contrasts with the general view that M2‐biased macrophages serve beneficial roles in muscle health, based on their demonstrated functions in increasing muscle repair and growth following increased muscle use or acute injury (Tidball & Wehling‐Henricks, 2007; Wang et al., 2014); those observations provided the basis for classifying M2‐biased macrophages as “reparative” or “pro‐regenerative” macrophages. At least in part, the beneficial effects of M2‐biased macrophages on muscle repair and regeneration are mediated by deactivating proinflammatory M1‐biased macrophages and releasing factors such as IGF1 and Klotho that directly promote myogenesis (Dumont & Frenette, 2010; Tonkin et al., 2015; Wehling‐Henricks et al., 2018). However, our current findings show that M2‐biased macrophage function in aging muscle is more similar to their role in chronic disease, at least with regard to muscle fibrosis. For example, the chronic muscle wasting disease that occurs in the *mdx* mouse model of Duchenne muscular dystrophy involves progressive muscle fibrosis that is accompanied by an accumulation of intramuscular M2‐biased macrophages that express TGFβ and arginase (Vidal et al., 2008; Villalta et al., 2009) and the ablation of arginase expression causes large reductions in the pathological fibrosis of dystrophic skeletal muscles (Wehling‐Henricks et al., 2010).

We were also surprised that myeloid‐specific mutation of *Spi1* selectively reduced numbers of CD163+ and CD206+ macrophages biased to the M2 phenotype in aging muscle without affecting the numbers of CD68+ macrophages, which would include macrophages biased toward the M1 phenotype. Furthermore, we observed that the *Spi1* mutation reduced the proportion of CD68+ macrophages that expressed the M2 phenotype marker, CD163. Together, those data indicate that *Spi1* mutation reduced macrophage activation to the M2‐biased phenotype, without reducing macrophage numbers. Previous investigations using animal models with conditional null or hypomorphic alleles of *Spi1* showed that ablating or decreasing *Spi1* expression at different stages of myeloid lineage determination can cause several defects in myeloid cell development (DeKoter et al., 2007; Rosenbauer et al., 2004), although effects on macrophage phenotype are rarely reported. However, closer examination of published literature shows that perturbations of PU.1 expression can influence the phenotype of macrophages in other pathological tissues. For example, a conditional PU.1‐deficient mouse line displayed attenuated allergic airway inflammation and reduced gene expression of the M2 macrophage markers *Ym‐1* and *Retnla* in lung tissues following exposure to allergens (Qian et al., 2015); this suggested that *Spi1* expression plays a significant role in alternative activation of macrophages to an M2 phenotype in that respiratory disease. Previous investigators also demonstrated that a population of Ly6C^hi^CD115^+^ monocytes that expressed high levels of PU.1 differentiate into monocyte‐derived dendritic cells in a GM‐CSF‐dependent manner, while a PU.1^lo^ subpopulation of Ly6C^hi^CD115^+^ monocytes differentiated into iNOS+ M1 macrophages upon microbial stimulation (Menezes et al., 2016). Those findings also suggest a link between levels of PU.1 expression and myeloid cell phenotype specification. Furthermore, *Spi1*+/− mice had reduced numbers of blood CD115^+^Ly6C^lo^ cells when presented with inflammatory stimulation and *Spi1*+/− mice generated iNOS+ macrophages in the spleen more efficiently than wild‐type mice (Menezes et al., 2016). These observations are generally consistent with our observations in our *Spi1*‐mutant mice, which indicate that decreasing, but not eliminating, the expression of *Spi1* in myeloid cells reduces M2 polarization and promotes M1 polarization of macrophages, without preventing the differentiation of hematopoietic stem cells into the myeloid lineage.

Another particularly intriguing observation in our present study is that the phenotypes of BMDMs in vitro and macrophages in muscles were affected differently by myeloid cell‐specific mutation of *Spi1*. Although intramuscular macrophages showed a specific reduction in M2‐biased macrophages, BMDMs from *Spi1*‐mutant mice showed no differences in M2‐macrophage‐related genes compared to floxed‐control BMDMs. These differences in effects of the mutation in muscle and in vitro may be attributable to the different environments in which these macrophages developed and were activated. BMDMs were cultured in vitro with recombinant M‐CSF to drive them towards macrophage differentiation, while intramuscular macrophages in aging muscle were activated in a more complex inflammatory environment. An important next step in future investigations will be to determine whether the differences between mechanisms that regulate macrophage phenotype in vitro and in aging muscle in vivo, as we report here, are specific to macrophage activation in the aging muscle microenvironment or regulate macrophage function in multiple, aging tissues throughout the body.

We also observed that the response of *Spi1*‐mutant BMDMs to forced M2 polarization with IL‐4 and IL‐10 treatment did not differ from the response of floxed‐control BMDMs. However, a previous investigation showed that *Spi1* ^+/−^ BMDMs displayed a blunted response to IL‐4 treatment in M2‐related gene expression (Qian et al., 2015). A major difference between these two models is that the *Spi1* mutation in our model is driven by *LysM* ^*Cre*^, whereas *Spi1* ^+/−^ BMDMs have reduced *Spi1* expression throughout all stages of hematopoiesis. These observations suggest that reducing *Spi1* levels at different stages of hematopoiesis affects the response of macrophage to M2 polarization.

More broadly, our findings which show that the influence of modifying *Spi1* expression on macrophage phenotype specification differs in vitro and in muscle in vivo further illustrates the limitations of projecting in vitro findings concerning regulation of macrophage phenotype to predict regulatory mechanisms in vivo (Murray et al., 2014). This limitation has also been demonstrated with regard to the transcriptional regulation of macrophage phenotype in injured skeletal muscle vs. in vitro models. For example, mutation of the transcription factor peroxisome proliferation activator receptor delta (PPARδ) reduces the expression of several M2 associated genes, including *Arg1*, *Mrc1* and *Retnla* in macrophages in vitro (Welc et al., 2020). However, *Ppard* mutation in macrophages in injured, inflamed muscle increases the expression of *Arg1* and *Retnla*, showing the inadequacy of projecting in vitro mechanisms that regulate expression of macrophage phenotypic markers to the more complex in vivo environment. These discrepancies between in vitro and in vivo observations concerning regulation of gene expression in macrophages may reflect to an unknown extent the heterogeneous origins of muscle‐resident macrophages (Wang et al., 2020).

As with other investigations using mouse models to study complex in vivo mechanisms, the extent to which the findings presented here pertain to the regulatory roles on macrophages in age‐related changes in human muscles will need to be tested experimentally. The genomic response of human tissues to trauma or disease that involves an inflammatory response shows poor correspondence to the genomic response in mouse models of trauma in which inflammation occurs. For example, changes in gene expression in human tissues experiencing acute trauma, burn, sepsis or infection showed little correlation to changes in gene expression in mouse tissue subjected to the similar, acute inflammation (Seok et al., 2013). The extent to which the murine immune response to muscle aging resembles the human response has not been examined in detail.

Although further investigations are needed to uncover the process of *Spi1* regulation of macrophage polarization and phenotype specification in complex, in vivo environments, our findings identify a previously unrecognized tool for directly targeting macrophage phenotype to prevent muscle aging. Our present study further illustrates the important regulatory roles played by M2‐biased macrophages in promoting sarcopenia and the accumulation of connective tissue in aging muscle. Together, these observations advance our understanding of the relationship between aging of the immune system and muscle aging.

## 4 EXPERIMENTAL PROCEDURES

### 4.1 Ethical approval

Guidelines provided by the Chancellor's Animal Research Committee at the University of California, Los Angeles (UCLA) were followed for all animal care and experimentation. All protocols involving the use of mice were conducted according to the National Institutes of Health Guide for the Care and Use of Laboratory Animals and were approved by the UCLA Animal Care and Use Committee (Animal Welfare Assurance number A‐3196).

### 4.2 Mice

*LysM* ^*Cre*^ mice (B6.129P2‐*Lyz2* ^*tm1[cre]Ifo*^/J) and *Spi1* ^flox^ mice (B6.Cg‐*Spi1* ^*tm2Dgt*^/J) were purchased from Jackson Laboratories (Bar Harbor, ME, USA) and bred in a specific pathogen‐free vivarium at the UCLA. Following euthanasia by inhalation of isoflurane, muscles were collected and frozen for sectioning and histological evaluation or frozen in liquid nitrogen until used for RNA isolation. Experimental groups included from 5–8 male mice per group.

### 4.3 RNA isolation and quantitative PCR

Muscles were homogenized in Trizol (Invitrogen) and RNA extracted, isolated and DNase‐treated using RNeasy spin columns according to the manufacturer's protocol (Qiagen). RNA was electrophoresed on 1.2% agarose gels and RNA quality assessed by determining 28S and 18S ribosomal RNA integrity. Total RNA was reverse transcribed with Super Script Reverse Transcriptase II using oligo dTs to prime extension (Invitrogen) to produce cDNA. The cDNA was used to measure the expression of selected transcripts using SYBR green qPCR master mix according to the manufacturer's protocol (Bio‐Rad). Real‐time PCR was performed on an iCycler thermocycler system equipped with iQ5 optical system software (Bio‐Rad). Reference genes were chosen following previously described methods (Wang et al., 2015). Based on that analysis, *Rps4x* and *Srp14* were used as reference genes for QPCR experiments. The normalization factor for each sample was calculated by geometric averaging of the *Ct* values of both reference genes using the geNorm software. Primers used for QPCR are listed in Table S1.

### 4.4 Immunohistochemistry and quantification of positive cells

Frozen cross‐sections were cut from the mid‐belly of quadriceps or TAs at a thickness of 10 μm. The sections were air‐dried for 30 min and fixed in ice‐cold acetone for 10 min, and endogenous peroxidase activity was quenched with 0.3% H_2_O_2_. Sections were blocked in 3% bovine serum albumin (BSA) and 2% gelatin in 50 mM Tris buffer (pH 7.2) for 30 min and then immunolabeled with primary antibodies for 3 h at room temperature anti‐PU.1 (eBioscience #14–9819), anti‐CD68 (Bio‐Rad #MCA1957), anti‐CD163 (Santa Cruz #SC33560), anti‐CD206 (Serotec #MC2235), anti‐collagen type I (Southern Biotech #1310–01), anti‐collagen III (Southern Biotech #1330–01) and anti‐MyoD (Santa Cruz #SC760). Antibodies to Pax7 were obtained from conditioned media from Pax7 hybridoma cells (Developmental Studies Hybridoma Bank) that were cultured in complete medium consisting of Dulbecco's Modified Eagle Medium (DMEM) with 1% penicillin–streptomycin (Gibco) and 20% heat‐inactivated fetal bovine serum (FBS). Antibodies to Pax7 were isolated from the conditioned medium as described previously (Wang et al., 2015).

Sections labeled with primary antibodies were washed with a phosphate buffered saline solution (PBS) and then probed with biotin‐conjugated secondary antibodies (Vector Laboratories) for 30 min. Sections were washed with PBS and then incubated for 30 min with avidin D‐conjugated horseradish peroxidase (Vector). Staining was visualized with the peroxidase substrate 3‐amino‐9‐ethylcarbazole (AEC kit; Vector), yielding a red reaction product. The numbers of immunolabeled cells in each section were counted using a bright‐field microscope and expressed as the number of cells/unit volume of each section. The volume of total muscle and the volume of muscle that consisted of connective tissue was determined using a stereological, point‐counting technique to determine section area and then multiplying that value by the section thickness (10 μm). Pax7+ cells/100 fibers and MyoD+ cells/1000 fibers were determined on entire cross‐sections of each muscle analyzed.

### 4.5 Peripheral blood leukocytes counting

Whole blood was collected from a femoral bleed. Red blood cells were lysed with ACK lysis buffer (Biowhitaker) that had been precooled on ice for 5 min. Blood samples were washed with Dulbecco's phosphate buffered saline (DPBS; Sigma) and then centrifuged for 5 min in clinical centrifuge at 1000 rpm. Pelleted cells were resuspended in 1 ml of DPBS and 200 μl aliquots of resuspended cells were centrifuged onto microscope slides using a Shandon 3 cytofuge for 5 min at 380 rpm. Samples were then rinsed briefly in DPBS, fixed with 2% formaldehyde solution for 5 min and stained with hematoxylin for 10 min. Slides were rinsed with distilled water and cell counts were performed using standard morphological criteria (O'Connell et al., 2015).

### 4.6 Bone marrow cell isolation and colony‐forming assay

BMCs were aseptically flushed from femurs and tibias of 12‐month‐old mice with DPBS and treated with ACK lysing buffer to clear red blood cells. Following a DPBS wash and filtration through a 70 μm filter, BMCs were counted with a hemocytometer and were resuspended in DPBS at the concentration of 2.5 × 10^6^ cells/ml. BMCs were cultured in methylcellulose medium 1.0% methylcellulose (Stemcell), 30% fetal calf serum (Gibco), 10 μM 2‐mercaptoethanol (Sigma), 1.0% L‐glutamine (Gibco), 1.0% sodium pyruvate (Gibco), 1.0% MEM vitamin solution (Gibco), 1.0% non‐essential amino acids (Gibco), 20 ng/ml GM‐CSF (Biosource), 10 ng/ml IL‐3 (Invitrogen), 10 ng/ml IL‐6 (Gibco) and 10 mg/ml stem cell factor (Gibco) in minimum essential medium (Corning Cellgro). BMCs were added to the methycellulose medium at a final cell density of 2 × 10^5^ cells/ml. The methycellulose medium containing BMCs was then vortexed and spun at 1000× *g* for 5 min. The methycellulose medium containing BMCs was then added to 3.5 cm culture dishes at 1 ml/dish and cultured at 37°C with 5% CO_2_ for 11 to 14 days. The number of colonies was then counted under a stereomicroscope.

### 4.7 Flow cytometry

BMCs were isolated as described above. Cells were then incubated in anti‐CD16/CD32 for 10 min to block Fc receptor binding. HSCs were then identified using combinations of antibodies conjugated to FITC, PE, PerCP/cy5.5 or Pacific Blue, as described previously (Table S2; Montecino‐Rodriguez et al., 2016). The lineage cocktail of antibodies included CD45R(B220), CD3ε, Gr‐1, IgM, NK1.1, TCRβ, TCRγδ and TER‐119. My‐HSCs and lymphoid‐biased HSCs (Ly‐HSCs) were identified within the lineage negative, CD48‐ population using antibodies to CD117(c‐Kit), CD135, CD150 and Sca‐1 (Figure 2d). GMPs, CMPs and MEPs in the lineage negative population were identified using antibodies to CD16/CD32, CD34, CD117 and Sca‐1 (Figure 2g).

### 4.8 Bone marrow‐derived macrophages culture and M2 polarization

BMCs isolated as described above were seeded at 5 × 10^6^ per 6‐cm dish in RPMI‐1640 (Sigma) with 20% heat‐inactivated FBS (Omega Scientific), penicillin (100 U/ml; Gibco), streptomycin (100 μg/ml; Gibco) and 10 ng/ml M‐CSF (R&D) at 37°C with 5% CO_2_ for 6 days. BMDMs were then stimulated for 6‐h with activation media consisting of DMEM with 0.25% heat‐inactivated FBS, penicillin, streptomycin and 10 ng/ml M‐CSF with or without IL‐4 (25 ng/ml; Sigma) and IL‐10 (10 ng/ml; Sigma). RNA was collected in Trizol for QPCR analysis as described above. PPIA and TPT1 were used as reference genes for QPCR experiments using BMDMs.

### 4.9 Immunocytochemistry on bone marrow‐derived macrophages

BMCs isolated as described above were seeded at 5 × 10^6^ per 6 cm dish in dishes containing sterile glass coverslips coated with 0.01% collagen, type 1 and 2% gelatin. BMCs were maintained in RPMI‐1640 (Sigma) with 20% heat‐inactivated FBS, penicillin (100 U/ml), streptomycin (100 ug/ml) and 10 ng/ml M‐CSF at 37°C with 5% CO_2_ for 6 days. BMDMs were then stimulated for 24 h with activation media consisting of DMEM with 0.25% heat‐inactivated FBS, penicillin, streptomycin, and 10 ng/ml M‐CSF. Coverslips were then fixed with 4% paraformaldehyde for 10 min, washed with PBS, and blocked in 3% BSA and 2% gelatin in 50 mM Tris buffer (pH 7.2) for 1 h. Coverslips were then immunolabeled with anti‐PU.1 for 3 h. Coverslips labelled with anti‐PU.1 were washed with PBS and probed with biotin‐conjugated secondary antibodies (Vector) for 30 min. Coverslips were subsequently washed with PBS and incubated with avidin D‐conjugated horseradish peroxidase for 30 min. Staining was visualized with the peroxidase substrate 3‐amino‐9‐ethylcarbazole. The number of immunolabeled cells was counted using a bright‐field microscope and expressed as the percentage of positive cells per total number of cells.

### 4.10 Cross‐sectional area measurement

Frozen cross‐sections were cut from the mid‐belly of quadriceps femoris and tibialis anterior (TA) muscles at a thickness of 10 μm. Sections were then stained with hematoxylin for 10 min. The muscle fiber cross‐sectional area was measured for 500 fibers randomly sampled from complete cross‐sections using a digital imaging system (Bioquant).

### 4.11 Statistics

Data are presented as mean ± SEM. One‐way analysis of variance was used to test whether differences between 3 or more groups were significant at *p* < 0.05. Significant differences between groups were identified using Tukey's post hoc test. Comparisons of two groups of values were analyzed using the unpaired, two‐tailed *t*‐test.

## AUTHOR CONTRIBUTIONS

Ying Wang, Steven S. Welc, Michelle Wehling‐Henricks, Enca Montecino‐Rodriguez, and James G. Tidball conceived and designed the experiments. Ying Wang, Steven S. Welc, Michelle Wehling‐Henricks, Ying Kong, Connor Thomas and James G. Tidball performed the experiments. Ying Wang, Steven S. Welc, Michelle Wehling‐Henricks, Ying Kong, Enca Montecino‐Rodriguez, Kenneth Dorshkind and James G. Tidball analyzed the data. Ying Wang, Steven S. Welc, and James G. Tidball wrote the initial draft of the manuscript. Ying Wang, Steven S. Welc, Michelle Wehling‐Henricks, Ying Kong, Enca Montecino‐Rodriguez, Kenneth Dorshkind, Connor Thomas and James G. Tidball edited and approved the final manuscript.

## CONFLICT OF INTEREST

The authors declare that they have no conflicts of interest.

## Supporting information


**Supplemental Figure 1** CD68+, CD163+, and CD206+ macrophages in aging skeletal muscle are not distinct macrophage subpopulations. (a) Quadriceps muscle cross‐section from a 22‐month‐old floxed‐control mouse stained for CD68 (488 nm; green) and CD163 (594 nm; red). Arrow shows double‐labeled cell (yellow). Arrowhead indicates a CD163 expressing cell that did not express detectible CD68. Bars = 25 μm. (b) A smaller proportion of CD68+ cells expressed CD163 in *Spi1*‐mutants than in floxed‐control muscles in 22‐month‐old mice. * indicates significant difference between genotypes at *p* < 0.05. *N* = 5 per data set. (c) Quadriceps muscle cross‐section from a 22‐month‐old floxed‐control mouse stained for CD206 (488 nm; green) and CD163 (594 nm; red). Arrow shows double‐labeled cell (yellow). Arrowhead indicates a CD163 expressing cell that did not express detectible CD206. Bars = 25 μm.

**Supplemental Table 1** Primers used for QPCR.
Supplemental Table 2. Antibodies used for FACS analysis.

## Data Availability

The data that support the findings of this study are available from the corresponding author upon reasonable request.
