# Supplementary figures and images for: Myeloid cell‐specific mutation of Spi1 selectively reduces M2‐biased macrophage numbers in skeletal muscle, reduces age‐related muscle fibrosis and prevents sarcopenia
Source: Aging Cell. 2022 Sep 13;21(10):e13690. doi: 10.1111/acel.13690 (PMC9577952; doi:10.1111/acel.13690)

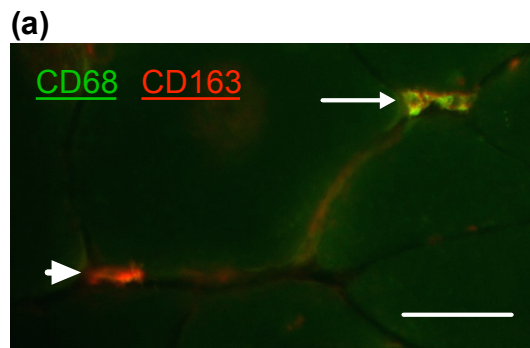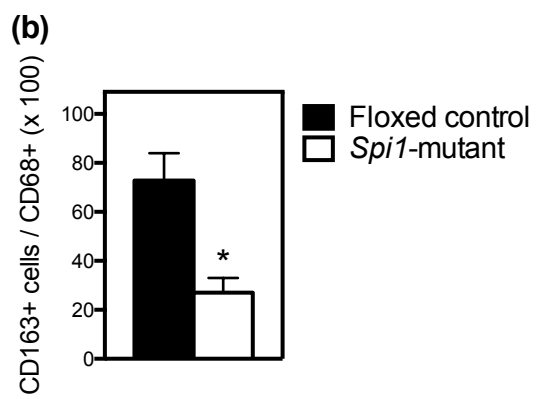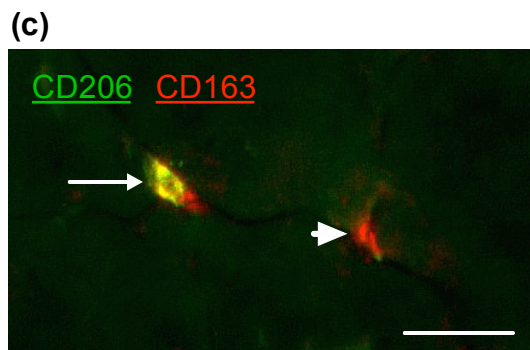

Supplemental figure 1.

Supplement: Supplementary file 1 — Supplemental Figure 1 CD68+, CD163+, and CD206+ macrophages in aging skeletal muscle are not distinct macrophage subpopulations. (a) Quadriceps muscle cross‐section from a 22‐month‐old floxed‐control mouse stained for CD68 (488 nm; green) and CD163 (594 nm; red). Arrow shows double‐labeled cell (yellow). Arrowhead indicates a CD163 expressing cell that did not express detectible CD68. Bars = 25 μm. (b) A smaller proportion of CD68+ cells expressed CD163 in Spi1‐mutants than in floxed‐control muscles in 22‐month‐old mice. * indicates significant difference between genotypes at p < 0.05. N = 5 per data set. (c) Quadriceps muscle cross‐section from a 22‐month‐old floxed‐control mouse stained for CD206 (488 nm; green) and CD163 (594 nm; red). Arrow shows double‐labeled cell (yellow). Arrowhead indicates a CD163 expressing cell that did not express detectible CD206. Bars = 25 μm. [file ACEL-21-e13690-s001.pdf]
