# Supplementary material for: Myeloid cell‐specific mutation of Spi1 selectively reduces M2‐biased macrophage numbers in skeletal muscle, reduces age‐related muscle fibrosis and prevents sarcopenia
Source: Aging Cell. 2022 Sep 13;21(10):e13690. doi: 10.1111/acel.13690 (PMC9577952; doi:10.1111/acel.13690)
Supplement: Supplementary file 2 — Supplemental Table 1 Primers used for QPCR. Supplemental Table 2. Antibodies used for FACS analysis. [file ACEL-21-e13690-s002.docx]

| Gene | Accession Number |  | Direction (5’->3’) |
| --- | --- | --- | --- |
| Arg1 | NM_007482 | Fwd | CAATGAAGAGCTGGCTGGTGT |
|  |  | Rev | GTGTGAGCATCCACCCAAATG |
| *Cd68* | NM_001291058.1 | Fwd | CAAAGCTTCTGCTGTGGAAAT |
|  |  | Rev | GACTGGTCACGGTTGCAAG |
| *Cd163* | NM_053094.2 | Fwd | GCAAAAACTGGCAGTGGG |
|  |  | Rev | GTCAAAATCACAGACGGAGC |
| *Mrc1 (CD206)* | NM_002438 | Fwd | GGATTGTGGAGCAGATGGAAG |
|  |  | Rev | CTTGAATGGAAATGCACAGAC |
| *Col1a1* | NM_007742.3_ | Fwd | TGTGTGCGATGACGTGCAAT |
|  |  | Rev | GGGTCCCTCGACTCCTACA |
| *Col3a1* | NM_009930.2 | Fwd | ATCCCATTTGGAGAATGTTGTGC |
|  |  | Rev | GGACATGATTCACAGATTCCAGG |
| *Retnla (Fizz1)* | NM_020509 | Fwd | TCGTGGAGAATAAGGTCAAGG |
|  |  | Rev | GGAGGCCCATCTGTTCATAG |
| *Myod1* | NM_010866.2 | Fwd | GAGCGCATCTCCACAGACAG |
|  |  | Rev | AAATCGCATTGGGGTTTGAG |
| *Pax7* | NM_011039.2 | Fwd | CTCAGTGAGTTCGATTAGCCG |
|  |  | Rev | AGACGGTTCCCTTTGTCGC |
| *Rps4x* | NM_009094.1 | Fwd | TGCTGGGTTTATGGATGTCA |
|  |  | Rev | CCTCCTCCGGTGTAATACGA |
| *Sfpi1* | NM_011355.2 | Fwd | CTACTCCTTCGTGGGCAGC |
|  |  | Rev | CTCTGCAGCTCTGTGAAGTGG |
| *Srp14* | NM_009273.4 | Fwd | AGAGGCGAGCAGTTCCTGAC |
|  |  | Rev | CGGTGCTGATCTTCCTTTTC |

Supplemental Table 1. Primers used for QPCR.

Antibody Clone Conjugate Source _______________________________________________________________________

Fcγ blocking CD16/32 93 eBioscience

________________________________________________________________________

| Lineage cocktail | CD45R |  | RA3-6B2 |  | FITC |  |  |  | eBioscience |
| --- | --- | --- | --- | --- | --- | --- | --- | --- | --- |
|  | CD3ε |  | 145-2C11 |  | FITC |  |  |  | eBioscience |
|  | CD48 |  | HM48-1 |  | FITC |  |  |  | Biolegend |
|  | Gr-1 |  | RB6-8C5 |  | FITC |  |  |  | eBioscience |
|  | IgM |  |  |  | FITC |  |  |  | Southern Biotech. |
|  | NK1.1 |  | PK136 |  | FITC |  |  |  | eBioscience |
|  | TCRβ |  | H57-597 |  | FITC |  |  |  | eBioscience |
|  | TCRγδ |  | UC7-13D5 |  | FITC |  |  |  | eBioscience |
|  | TER-119 |  | TER119 |  | FITC |  |  |  | eBioscience |

________________________________________________________________________ HSCs CD117 ACK2 APC eBioscience CD135-biotin A2F10 avidin/Pacific Blue eBioscience

CD150 TC15-12F12.2 PE Biolegend

Sca-1 D7 PerCP/cy5.5 eBioscience ________________________________________________________________________ CMPs/GMPs/MEPs CD16/32 93 PE eBioscience

CD34 RAM34 Pacific Blue eBioscience

CD117 ACK2 APC eBioscience

Sca-1 D7 PerCP/cy5.5 eBioscience

Supplemental Table 2. Antibodies used for FACS analysis.
